# Pandemic Preparedness: What Difference Does Experience Make?

**DOI:** 10.1017/S0021932025000185

**Published:** 2025-04-11

**Authors:** Marion Baby-May Nyakoi

**Affiliations:** Njala University, Bo, Sierra Leone and Eastern Technical University, Kenema, Sierra Leone

I carried out ethnographic research for the Pandemic Preparedness Project in NG and its satellite settlements in chiefdom in Kailahun District, Eastern Province of Sierra Leone. NG has a Peripheral Health Unit (PHU) which provides health services to seven villages in the section and to one other, which is a long distance from its designated facility. The research location attracted our attention as a research site, as it was one of the first and worst affected communities during the 2014-16 Ebola outbreak in Sierra Leone. The work aimed to understand communities’ own pandemic preparedness, using a long term observational research method. This means that the research was residence based, during which the researcher becomes an observer very well known to the community and becomes accustomed to local ways of life. The ethnographic work I undertook aimed to understand the health-seeking behaviour of local people in the aftermath of a major challenge (Ebola). The Covid-19 pandemic began while I was in the field, so the question of how people understood and reacted to this second infection challenge became central to the study. Sierra Leone has very few female social scientists trained in using ethnographic approaches. I was trained in Nutrition and Dietetics, both as an undergraduate and postgraduate. Doing ethnographic work was a shock to me and these notes are intended to help other female researchers understand better what it might be involved in understanding ethnographic methods to throw light on bio-social data.

I started ethnographic research in NG in September, 2020. The community found my arrival a threat in the sense that I am an educated person, coming from Eastern Technical University (the then Eastern Polytechnic Technical College) in Kenema, a spy, seeking to know about their livelihood activities and health thus as one informants told me “Buwai nai mu gubyEi vaa” (you have come to watch us) when paying a visit to a household. The community people thought that I was a journalist and had come to collect information from them and take this information to the government. The young ladies were suspicious and worried for their husbands, having in mind that I was a young beautiful and educated woman. Therefore, they thought of their husbands making advances at the researcher and the tendency of the researcher yielding to those husbands and creating misfortunes in their homes. I made them relaxed and changed their thoughts by getting closer to the young ladies. After months of being with them, they saw that they did not face any misunderstandings or have any complaints but saw the researcher as somebody that was serious and just focused on her work.

Also, to ease their apprehensions, during a long stay, I ‘adopted’ a local godfather and local godmother to guide and protect me from any unintended misbehaviour, and to resolve disputes between me and others in the communities. I also established friendships with one or two women I took with me, accompanied by their children, to visit other communities in the region. Thus like all other village residents I now have a village family, and this helped to normalize my presence. So as time passed, I began to be seen as one of them and this allowed people to unveil facts about medicine and about Ebola response they might otherwise have chosen to keep hidden. With consent, I used my phone and voice recorder to back-up my observational studies without causing fear or comments. Most people have still clear memories about Ebola in the communities. One informant put it this way: “I am just living with the help of God. The day my parents died was the end of everything”. I decided to try getting a comprehensive background on how and why so many died in one village during the epidemic.

The Ebola outbreak was shocking and well-remembered. I did some interviews around Ebola. On the 23^rd^ May, 2014 a nurse working in Kissi Tengbeh chiefdom fell ill with an unknown fever, and was referred to the Kenema hospital Lassa Fever ward directly. According to the deceased brother KB he explained that “On their way to Kenema her husband thought it good to take his wife to their own health centre in D, since both were from the same chiefdom, and she was now very ill”. According to normal local family responsibilities concerning illness the husband then had to inform relatives about his wife’s sickness and condition. So, he informed the town chief of her village. He then sent messages to various people in NG and surrounding villages about the nurse’s sickness and poor condition. As a result many concerned people came right away to visit, to sympathize, to feed and clean her in the hospital. These visitors included the wife of the Paramount Chief and head of the PHU at NG. However, her condition worsened and she died a day after admission, and was buried in her husband’s village. A three day ceremony was then held in NG. But at this time some people began to show symptoms of the fever. On 28^th^ May 2014 six people were taken to the PHU in NG, but it was now clear that an outbreak of something unknown was starting, and these people were sent or taken directly to the government hospital in Kenema, where three of them died, including the in-charge of the PHU at NG. People in these rural areas are highly connected by family or marriage so whenever a family member was infected or died of the disease other close connections attended the sickbed or funeral and the more cases were caused. In all eighty nine people in NG were infected in this outbreak, and sixty nine people died. People living through 2014-2015 Ebola crises were marked for life, and still talked about the devastating impact on their families. They reported that families were incomplete, at least one member of a household died of the disease, farm work was undermined, since labour supply depended on family exchanges. Ebola affected the people in many ways as the I was told by few of the people I interviewed. These included the loss of family members and some homes were left empty. Ebola caused the community not to be in unity, showing less concern than before; no visitation, even when somebody was sick nobody was allowed to visit, people died all by themselves and will not be noticed throughout that day as the people were banned from touching the sick. The road leading to other communities was avoided completely by travelers and other people from the nearby villages/communities. There was no food in the whole town. Farm work was totally interrupted as all the reserved rice for farming was eaten.

One aspect of ethnography is the need to keep an open mind to enquire into and make observations of anything that might turn out to be important. This has to have some practical limit, or there would be no time for more focused work later. Since my focus was on pandemic preparedness I made a check list of topics, and paid attention to everything that seemed relevant to as a result of the Ebola episode. These topics included worries about hygiene and sanitation (what is a virus and how does it spread?), worries about livelihoods, drinking water and diet (how will they cope under lockdown, for example). As I became familiar to people I faded into their background. I was on the above topics for months when Covid-19 struck the country, and I was now in a good position, as a well-assimilated observer, to witness how the people of NG coped with this new threat and what ways they might change their responses as the new pandemic unfolded.

Covid-19 was a shock to the inhabitants of Sierra Leone, and this changed our research focus from preparedness to response. I now had a standard for comparison. I was able to discuss with people in the community about Covid-19 relative to Ebola. There were immediate similarities. For a start, there were once again a need to adhere to strict lockdowns rules and byelaws to control infection (Kamara et al. 2022). At the beginning there were widespread understandings that the response for Ebola based on bylaws and quarantine would also protect against Covid-19. All social gatherings were banned, and hand washing, mask wearing, social distancing were imposed by the government. The experience of Ebola affected people as the response to Covid-19 was high. Bylaws were put into place and enforced. At the initial time no movement was allowed in and out of the community of Njala Giema especially when the signs of Covid-19 were read to them (community people). This was done as the community people had not wanted to go back to the experienced they had in the time of Ebola.

Covid-19 is harder to detect since it spreads in the air, has less dramatic symptoms and seriously affects mainly older people unlike Ebola, spreads through contact with body fluids and has more dramatic symptoms. Whether or not there were cases of Covid-19 in NG was hard to detect. But these regulations to protect against it brought back bad memories of how relatives had died of Ebola, and how the community had been cut off from all contact for many weeks. Stakeholders and community leaders rushed to revive local byelaws to make sure their people abided by the national and local rules and regulations to control the new virus. So, there was no visitation either by both strangers and even family members across community and neighborhood boundaries. Even residents from other towns in the chiefdom had first to visit the local clinic to be screened for Covid-19 by a community health officer. Some military personnel even paid regular visits to my area of work. Why they came is not clear. They seemed to assume that if this area had had a high Ebola death rate it must have a high death rate from Covid-19, and that iron discipline was the way to avoid a further high death toll. Most people complained about these restrictions. My field notes record one informant summing this up in the following words: “we have been restricted again; no schools, no mosque worship, no *ndowei* [periodic market], no touching, and we have not even seen any case of Covid-19”. Invisibility is not the only problem with Covid-19. The disease is mainly life threatening among older people, and from my household censuses in NG it was clear that older people were only a small portion of total population see Table 1 below:

Almost all goods other than locally produced food stuffs come from Freetown down to the last village in Sierra Leone. The lockdown and travel restrictions had major impacts on supply chains and food security, since all markets were closed and imported food stuffs like white rice (an import in the rainy season, since most people farm either upland rice or crops like cacao and coffee), salt, fish, etc. were unavailable. Wherever supplies were available prices increased.

In a crisis, children often experience significant social or economic disadvantage impacting seriously on their care or development (particularly in communities with existing poor health care). With that in mind, my work focused on the nutritional status of children. As restrictions eased, I discussed with mothers some of the challenges they had faced during lockdown. One issue was that mothers had reduced access to enough appropriate food for their children. One female remarked: “*gOmEtei yEi mu O hEga*i *kovidi va* (‘the government told us to sit down due to the Covid-19 outbreak’). I enquired in general about weaning foods for children, and discussions showed that most mothers introduced weaning foods to support breast milk as early as three months. From this time onwards some mothers feed their babies with either ‘rice porridge’ (pounded rice blended with a drop of palm oil and a pinch of salt) or others make ‘local porridge’ (by mixing glucose biscuit with ‘rice porridge’). In lockdown the biscuits were unavailable so these mothers only offered ‘rice porridge’ for their babies. My field notes record that ‘even if you had money to buy glucose biscuit to mix with the rice flour for your child it is not possible because of lockdown’ (10^th^ March, 2022). Where they could, mothers exchanged food items like salt, dried fish, palm oil or cooked foods among themselves. They also undertook garden work to grow items they could no longer buy. They also helped each other to harvest and process red palm oil and gari (cassava meal); those assisting would then expect to receive a portion to sustain her family – as one mother reported in Creole “ Tel God tenki for de fod dem lek cassada, petehteh, pepeh,etc. wae dae hipi wii sef wit de fod wii pul na wii gardin foi kep wii fambul dem “ (‘thanks be to God for the foods like cassava, sweet potatoes, pepper, etc. we get from each other harvesting our garden items to sustain our families’) as one mother reported.

Some people found ways to get bush yams, cassava, potatoes, or anything edible from their bush and farms during the lockdowns. Given the general restriction of movement most people told me that Covid-19 was worse than Ebola since under Covid-19 restrictions it were much harder to find ways to provide food for their families. One man told me “now our wives cannot do most activities that earned money such as fishing, farming, petty trading, garri making, thrift, and labour cooperation are all at a standstill”. I then turned my focus on lockdowns by observing the impacts of it on normal activities by farmers’ bike riders, and livelihood in general in NG and in its satellite villages

Covid-19 is an airborne disease. Mask wearing was one of the rules for Covid-19 prevention. I did not see much mask wearing. People told me that using face masks restrict them from breathing properly. On the other hand some bike riders realized that masks prevented them from inhaling dust while riding, and this set a standard others began to follow as one bike rider confirmed; “we are now using the mask more because it prevent us greatly from inhaling dust as the roads are mostly dusty in the dry season”. (Field note, August 2021, why do you wear facemask). I also asked about hand washing. One informant responded by saying: “You people brought us a veronica bucket, which at first we were using, but we got tired of washing hands; where is the food you brought us to eat? I do not have anything bad in my hands, my hands are clean, but we have no food. And as for social distancing, we do not have any stranger in our midst” (field note April, 2021). This is an important distinction about what people locally perceive to be urgently needed during Covid-19 and what should be provisioned to the local community to help them survive it. Further, the informant also suggests that social distancing is not necessary because there are no ‘strangers’ in the midst. This helps to contextualise how the community understood who is and is not a carrier of disease. I made a follow up visit to this informant. At that time, I was constantly asking about hand washing, the wearing of face masks, and social distancing. Being that people were under travel restrictions, and no strangers were allowed in the community, the people were confident of themselves and thought that there was no means for Covid-19 to affect them. Even when they started allowing people to come to their community, those people had to first visit the Peripheral Health Unit (PHU) for screening before they were allowed to stay in the community.

Vaccines began to become available in 2021. There were many rumours passing around about these vaccines about vaccine for Covid-19 and I duly took note of this build-up of rumours. Much of it came from overseas via people in Kenema who had links to social media. Some thought that vaccines were only ever taken by children. Adults had no need of them. Others had heard rumours that there was a Chinese campaign to reduce the population of Africa, and those vaccines were the way this was going to be done. Others were convinced that vaccines would produce serious side effects only after a number of years. Lots of people gave their opinions on vaccine anxiety. At first people were saying vaccine (Maklate) were for children but when Covid-19 vaccine came into the country and the community people saw lots of stakeholders, prominent people taking the vaccine, then few started to take it. Good testimonies of people in the community that took the vaccine while in big towns made it possible for the majority of the community to be willing to take the vaccine. Lots of testimonies like back pain being healed, menstruation flow started again after blockage for some months, good eyesight and many more followed.

Sierra Leone received its first Covid-19 vaccines (AstraZeneca and Sinopharm types) on 8th March 2021. On 22 March 2021 the president and his cabinet were filmed for TV having the first shots, followed the health and military personnel. AstraZeneca was for people with underlying medical conditions and the aged while the Chinese Sinopharm vaccine was made available to all age groups. I took my own first shot of the Sinopharm vaccine at Kenema Government Secondary school on 29 March 2021. Two vaccines were later administered in the country, made by Johnson & Johnson and Pfizer. The single shot vaccine made by Johnson & Johnson was given to people in NG and I was curious to observe the uptake for this vaccine.

No one was aware of any Covid-19 cases affecting the community. We have no means of knowing whether cases occurred because there was no community level testing. The only testing took place for staff of a nearby oil palm plantation. I was surprised that many people took the vaccine and then reported positive effects for other conditions. An old woman stated that “since I have received this vaccine, I have lost my long-term back pain”. A young woman claimed that “I stopped seeing my menstruation but it started coming again after taking the vaccine”. A youth in NG reported that “I was having difficulties in seeing clearly but now I can see even from afar”. Another said that “the cold that was disturbing me is gone”. I am now left with the puzzle of trying to understand these alleged cures, but they may explain why vaccine hesitation was not the problem we expected it to be. This kind of surprise is why ethnographic fieldwork is so useful.

Ethnography as a method is so helpful it gives researchers the privilege to interact, sit with people in many functions like meetings and helps the researcher to know and study more about the community people. This all helped the researcher to see and understand what is really happening and if it is the same as people told you it was. At times what people told you is different from what you will see when interacting in community life.

## Figures and Tables

**Table 1:** Average age of old people in NG

No	Community Name	Quarters	Average age 50+
1	NG	Five quarters	2 % were between the average age of 50+
